# Platelet Interactions with Liver Sinusoidal Endothelial Cells and Hepatic Stellate Cells Lead to Hepatocyte Proliferation

**DOI:** 10.3390/cells9051243

**Published:** 2020-05-18

**Authors:** Jeremy Meyer, Alexandre Balaphas, Pierre Fontana, Philippe Morel, Simon C. Robson, Karin Sadoul, Carmen Gonelle-Gispert, Léo Bühler

**Affiliations:** 1 Division of Digestive Surgery, University Hospitals of Geneva, 1205 Genève, Switzerland; Alexandre.balaphas@hcuge.ch (A.B.); pmorel@beaulieu.ch (P.M.); 2 Unit of Surgical Research, Medical School, University of Geneva, 1205 Genève, Switzerland; 3 Division of Angiology and Hemostasis, University Hospitals of Geneva, 1205 Genève, Switzerland; pierre.fontana@hcuge.ch; 4 Geneva Platelet Group, Medical School, University of Geneva, 1205 Genève, Switzerland; 5 Department Medicine, Beth Israel Deaconess Medical Center, Boston, MA 02215, USA; srobson@bidmc.harvard.edu; 6 Regulation and Pharmacology of the Cytoskeleton, Institute for Advanced Biosciences, Université Grenoble Alpes, 38400 Saint-Martin-d’Herès, France; karin.sadoul@univ-grenoble-alpes.fr; 7 Faculty of Science and Medicine, University of Fribourg, 1700 Fribourg, Switzerland; carmen.gonelle@unifr.ch (C.G.-G.); leo.buhler@unifr.ch (L.B.)

**Keywords:** LSEC, HSC, hepatocyte, IL-6, interleukin-6, HGF, liver regeneration

## Abstract

(1) Background: Platelets were postulated to constitute the trigger of liver regeneration. The aim of this study was to dissect the cellular interactions between the various liver cells involved in liver regeneration and to clarify the role of platelets. (2) Methods: Primary mouse liver sinusoidal endothelial cells (LSECs) were co-incubated with increasing numbers of resting platelets, activated platelets, or platelet releasates. Alterations in the secretion of growth factors were measured. The active fractions of platelet releasates were characterized and their effects on hepatocyte proliferation assessed. Finally, conditioned media of LSECs exposed to platelets were added to primary hepatic stellate cells (HSCs). Secretion of hepatocyte growth factor (HGF) and hepatocyte proliferation were measured. After partial hepatectomy in mice, platelet and liver sinusoidal endothelial cell (LSEC) interactions were analyzed in vivo by confocal microscopy, and interleukin-6 (IL-6) and HGF levels were determined. (3) Results: Co-incubation of increasing numbers of platelets with LSECs resulted in enhanced IL-6 secretion by LSECs. The effect was mediated by the platelet releasate, notably a thermolabile soluble factor with a molecular weight over 100 kDa. The conditioned medium of LSECs exposed to platelets did not increase proliferation of primary hepatocytes when compared to LSECs alone but stimulated hepatocyte growth factor (HGF) secretion by HSCs, which led to hepatocyte proliferation. Following partial hepatectomy, in vivo adhesion of platelets to LSECs was significantly increased when compared to sham-operated mice. Clopidogrel inhibited HGF secretion after partial hepatectomy. (4) Conclusion: Our findings indicate that platelets interact with LSECs after partial hepatectomy and activate them to release a large molecule of protein nature, which constitutes the initial trigger for liver regeneration.

## 1. Introduction

Acute liver failure is a medical emergency, which develops when liver function does not meet physiological requirements due to a critical loss of functional mass. This life-threatening syndrome occurs following drug toxicity, viral hepatitis, major liver resections, or small-for-size liver transplantation [1]. Techniques to prevent surgery-related liver failure in high-risk patients are mainly based on taking into account comorbid conditions, on limiting the extent of surgery, and on increasing the volume of the future remnant liver [2]. Despite these precautions, many patients, particularly those with liver cirrhosis, cannot benefit from liver resection as a therapeutic procedure. Further, whole liver transplantation currently remains the only option for patients who are unlikely to recover from acute liver failure with standard care [2]. It is therefore of main interest to understand how liver regeneration is regulated and what molecular factors stimulate the regenerative process.

In this context, platelet count was directly correlated to hepatocyte proliferation after partial hepatectomy in rodents [3]. Recently, perioperative platelet count has been identified as a predictor of post-operative liver function and mortality in cohorts of patients after partial hepatectomy [4], in cirrhotic but also in non-cirrhotic patients [5]. Moreover, the number of circulating platelets and platelet transfusion were described to correlate with liver volume increase after both partial hepatectomy [6] and living-donor liver transplantation [7], respectively. However, the mechanism by which platelets exert their beneficial effect on liver regeneration remains elusive.

Interactions between platelets and endothelial cells were shown to be determinant in several physiological and pathological processes, such as wound healing [8], angiogenesis [9], and ischemia-reperfusion [10]. Extracellular vesicles have been identified as key mediators of cell-to-cell communication in liver physiology, and platelet-derived extracellular vesicles are postulated to be involved in these interactions [11,12]. Further, human platelets were recently described to release, in response to distinct protease-activated receptor 1 (PAR1) or 4 (PAR4) stimulations, molecules with opposing effects on endothelial cells [13,14]. Recently, liver sinusoidal endothelial cells (LSECs) were shown to express angiogenic growth factor receptors after partial hepatectomy. Further, LSECs were demonstrated to regulate liver regeneration through angiocrine signaling [15].

Therefore, we postulated that platelets modulated the LSEC secretory response and could therefore constitute early paracrine trigger of liver regeneration [3,16]. In the present study, we thoroughly investigated the importance of interactions between platelets and non-parenchymal liver cells in terms of liver regeneration to provide new insight in the field.

## 2. Materials and Methods

The materials and methods are also reported in the Supplementary Materials.

### 2.1. Animals

C57BL/6 male mice aged 10–16 weeks were obtained from Janvier Labs (Le Genest-Saint-Isle, France). All procedures were performed in accordance with protocols approved by the animal ethics committee of the Geneva Veterinarian Office (GE27-15, GE127-15, GE16-74, GE37-18, GE 49-17, GE90-16, GE133-15, GE1043/3787/3) and the University of Geneva (Geneva, Switzerland). All mice were maintained under standard conditions at the animal facility of the University of Geneva.

### 2.2. Platelet Isolation and Activation

Blood was collected from C57BL/6 male mice by cardiac puncture into acid-citrate-dextrose solution and centrifuged at 100× *g* for 15 min to obtain platelet-rich plasma. Platelet-rich plasma was supplemented with 2.5 µL/mL PGI2 and centrifuged at 600× *g* for 15 min to obtain washed platelets. Washed platelets were counted on a KX-21N automated hematology analyzer (Sysmex, Horgen, Switzerland) and adjusted to 500,000 platelets/µL using Tyrode’s buffer (Figure S1A). Depending on experimental procedures, platelets were activated by platelet weak (adenosine diphosphate—ADP [17]) or strong (thrombin [17]) agonists (Figure S1B, left panel) 1 h after the addition of PGI2. Activation was also performed by PAR4 modulation using either a PAR4 agonist (AY-NH2) [13,14,18,19] (Figure S1B, left panel) or a PAR4 antagonist (TcY-NH2) in association with thrombin (14, 18) (Figure S1B, right panel). Selection of the concentrations of platelet agonists and antagonists were determined by titration using a TA-8V aggregometer (SD Medical, Heillecourt, France). The minimal concentrations of agonist/antagonist leading to 75% of maximal aggregation/inhibition were determined and then applied in experiments for platelet activation. Platelet releasates were prepared by adding 2.5 µL/mL PGI2 to activated platelets, which were pelleted at 600× g for 15 min. The supernatant was harvested and defined as the platelet releasate, whereas the pellet was considered as degranulated platelets and suspended in a similar volume of Tyrode’s buffer (Figure S1C).

### 2.3. Isolation and Co-Culture of LSECs or Macrophages with Platelets

LSECs and macrophages were isolated according to a method we have previously described (see Supplementary Materials) [20]. Briefly, 100,000 LSECs or 75,000 macrophages in culture for 12 h were rinsed, and 150 µL William’s Medium 1% FCS was added to each well. After 2 h, 0.16, 0.8, 3.2, or 16 million resting, activated platelets or platelet fractions were added to wells. The culture plates were centrifuged at 50× *g* for 30 s to spin down platelets on LSEC. Cells were then incubated at 37 °C for 24 h. Conditioned medium was harvested after 24 h.

### 2.4. Isolation and Culture of Hepatic Stellate Cells

Hepatic stellate cells (HSCs) were isolated according to a published method (see Supplementary Materials). Culture medium was changed after 12 h. Twenty-four hours after HSC isolation, LSEC conditioned medium or a solution of IMDM 10% FCS with or without recombinant interleukin-6 (IL-6) was added. Conditioned medium was retrieved after 48 h. To obtain conditioned medium for hepatocyte cultures, LSECs and HSCs were cultured in FCS-free medium with or without platelet releasate. Conditioned medium was harvested at 24 and 48 h, respectively. Hepatocytes were isolated and processed as described in the Supplementary Materials (Methods). Conditioned medium was added after 72 h in culture.

### 2.5. Statistical Analysis

Results were expressed as means ± standard error of the mean (SEM), if not indicated otherwise. Differences between groups or conditions were determined using the non-parametric Mann–Whitney rank-sum test, the t-test, or the paired t-test for ratios, when appropriate. Correlation analyses for continuous variables were performed using linear regression. The null hypothesis was rejected at *p* < 0.05. GraphPad Prism version 6 (GraphPad Software Inc, La Jolla, USA) and STATA version 13 (StataCorp LP, College Station, USA) were used for statistical analyses.

## 3. Results

### 3.1. Platelets Induce the Release of IL-6 from LSECs but Not from Resident Macrophages

**Figure 1:**
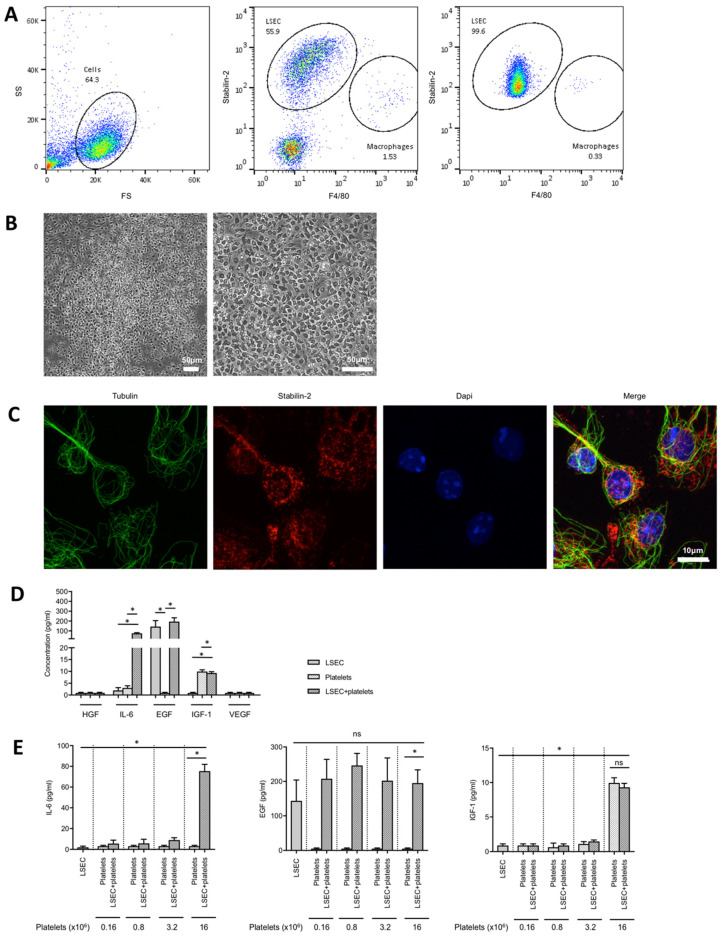
Platelets stimulate liver sinusoidal endothelial cells (LSECs) to release interleukin-6 (IL-6). (**A**) Dot-plots of flow cytometry analysis for CD11b-negative cells. Left panel: gating strategy. Middle panel: counting step, after CD11b-MACS but before long-term selective adherence. Right panel: purity after long-term selective adherence. LSECs were defined as stabilin-2+ F4/80- cells, macrophages as F4/80+ cells. Unstained cells were considered as unidentified. (**B**) Purified primary LSECs in culture 24 h after isolation. Left panel: magnification 100×. Right panel: magnification 200×. (**C**) LSECs were characterized by immunofluorescence using antibodies against stabilin-2 (red) and tubulin (green); nuclei are stained with dapi (blue). (**D**) LSECs were cultured for 24 h with or without 16 million resting platelets and release of growth factors (hepatocyte growth factor—HGF, IL-6, epidermal growth factor—EGF, insulin-like growth factor-1—IGF-1, and vascular endothelial growth factor—VEGF) was measured. (**E**) LSECs were co-cultured with different quantities of resting platelets (from 0.16 to 16 million), and releases of IL-6, EGF, and IGF-1 were measured in the culture medium. N = 4, * *p* < 0.05.

Interactions between platelets and LSECs were postulated to constitute the early trigger of liver regeneration [3,16]. To investigate the effects of contact between platelets and LSECs, confluent pure primary LSECs (Figure 1A–C) were cultured in the presence of increasing numbers of resting platelets, and the release of growth factors was measured by ELISA assays. Hepatocyte growth factor (HGF) and vascular endothelial growth factor (VEGF) were not detected in the co-culture medium at 24 h (Figure 1D and Figure S2). Epidermal growth factor (EGF) was released by LSECs, but this secretion was not modulated by platelets (Figure 1D,E). Platelets, but not LSECs, released insulin-like growth factor-1 (IGF-1) (Figure 1D,E and Figure S2).

Of note, platelets alone did not release IL-6, but potentiated paracrine IL-6 release from LSECs. For instance, IL-6 secretion tended to increase when 0.16, 0.8, and 3.2 million resting platelets were added to LSECs when compared to LSECs without platelets, but this result did not reach significance (Figure 1E). Incubation with 16 million resting platelets significantly increased IL-6 release from LSECs by approximately 40-fold compared to LSECs alone (Figure 1E). Further, IL-6 release was proportional to the amount of resting platelets added to LSECs when using linear regression with platelets set as the independent variable and IL-6 as the dependent variable (*b* = 4.56, 95% confidence intervals [3.94–5.18], *p* < 0.001, adjusted R^2^ = 0.93).

IL-6 secretion by liver resident macrophages (Kupffer cells) was shown to be required for liver regeneration [21]. Although working with highly pure LSECs, as previously shown [20], we wanted to determine whether resident macrophages could contribute to the observed platelet-induced IL-6 secretion. Therefore, we cultured isolated primary macrophages (Figure S3A, left panel) in the presence or absence of 16 million resting or activated platelets, and the results clearly showed that IL-6 secretion from macrophages was not modulated by adding resting or activated platelets (Figure S3A, right panel). To further confirm that LSECs release IL-6 in the absence of contaminating macrophages, we cultured LSECs, isolated from in vivo macrophage-depleted mice (Figure S3B, left panel), in the presence of resting or activated platelets. IL-6 secretion from LSECs was still significantly increased when platelets were added to LSECs (Figure S3B, right panel) and in a pattern similar to the results obtained from LSECs isolated from mice not depleted of macrophages. Therefore, resident macrophages did not play a role in the observed platelet-stimulated IL-6 secretion by LSEC.

### 3.2. IL-6 Released from LSECs upon Contact with Platelets Induces HGF Secretion from HSCs and Leads to Hepatocyte Proliferation

**Figure 2:**
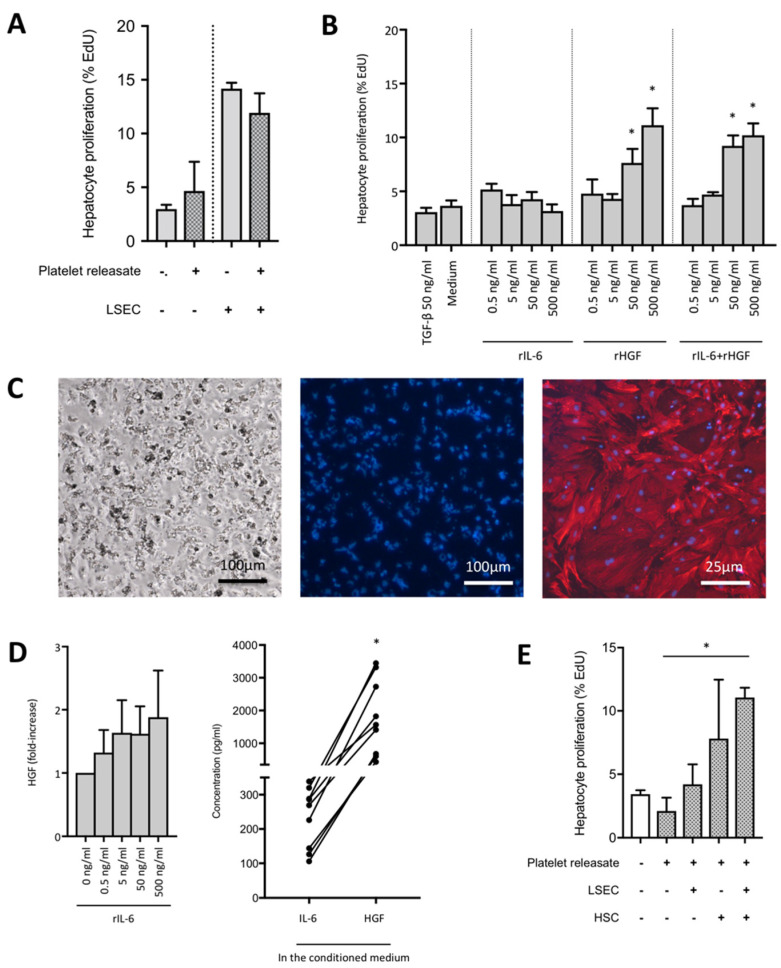
IL-6 released from LSECs upon the action of platelets stimulates HGF secretion from HSCs and leads to hepatocyte proliferation. (**A**) Hepatocyte proliferation according to EdU incorporation after 24 h in culture, in the presence or not of platelets and/or LSECs. N = 3. (**B**) Hepatocyte proliferation according to EdU incorporation, in the presence or not of increasing concentrations of recombinant IL-6 and/or HGF. N = 6, * *p* < 0.05. (**C**) Left: Autofluorescence of freshly isolated HSCs after excitation with an ultraviolet source. Middle: Oil-Red-O staining of HSCs cultivated 24 h. Magnifications 200× and 400×. Right: HSCs were stained with antibodies against α-SMA (red) after 7 days of culture following their activation with TGF-β1. Nuclei are stained with Hoechst (blue). (**D**) Left panel: HGF secretion at 24 h of culture of HSCs in the presence of increasing concentrations of recombinant IL-6. N = 3. Right panel: HGF secretion at 24 h of culture of HSCs exposed to the conditioned medium of LSECs cultured for 24 h with the releasate of activated platelets. Left dots: concentration of IL-6 in the conditioned medium, right dots: concentration of HGF released by HSCs when exposed to the conditioned medium. N = 9, * *p* < 0.05. (**E**) Hepatocyte proliferation according to EdU incorporation after 24 h in culture, in the presence or not of platelets, LSECs and HSCs or conditioned-medium of LSECs cultured with platelets or of HSCs cultured with the latest. N = 2–3 (depending on experimental groups).

Several molecules released from platelet α- and dense granules were reported to be involved in the direct proliferative effect of platelets on hepatocytes [3], but also, recent evidences suggested that hepatocyte proliferation could be induced by mRNA transfer from platelets to hepatocytes following platelet internalization [3,22]. However, we did not observe a proliferative effect of activated platelets or releasate from activated platelets on primary hepatocytes in culture (Figure 2A).

Of interest, IL-6 is a known inducer of liver regeneration in vivo, but its effects on liver regeneration remain debated [23]. To determine whether IL-6 induces hepatocyte proliferation in vitro, we cultured primary hepatocytes in the presence of recombinant IL-6 and/or HGF for 24 h. HGF had a strong mitogenic effect on hepatocytes, whereas IL-6 alone did not increase hepatocyte proliferation (Figure 2B). To investigate whether the platelet-induced IL-6 secretion from LSECs stimulates hepatocyte proliferation, we cultured primary hepatocytes with the conditioned medium of LSECs and platelets for 24 h. Conditioned medium from LSECs alone stimulated hepatocyte proliferation according to EdU incorporation, but an additional stimulating effect of supernatants of LSECs after platelet incubation was not observed (Figure 2A,E). Therefore, IL-6 alone is not sufficient to stimulate primary hepatocyte proliferation in vitro.

Thereafter, to determine whether interplay with a third actor of the liver sinusoid—HSC—is necessary to induce hepatocyte proliferation, we investigated the effect of recombinant IL-6 on HGF secretion from primary HSCs (Figure 2C), and we found a trend towards HGF secretion from HSCs (Figure 2D, left panel). Then, we investigated HGF secretion from cultured primary HSCs exposed or not to the conditioned medium of LSECs cultured or not with releasates of activated platelets. We found that the conditioned medium from LSECs cultured with releasates of activated platelets induced HGF secretion by HSCs (Figure 2D, right panel). HGF secretion by HSCs was directly proportional to the concentration of IL-6 contained in the conditioned medium of LSECs exposed to platelets when using linear regression with IL-6 concentration in the medium set as the independent variable and HGF concentration as the dependent variable (*b* = 5.48, 95% confidence intervals [2.28–8.68], *p* = 0.002, adjusted R^2^ = 0.31).

Finally, we cultured primary hepatocytes in the presence or not of the releasate of activated platelets, in the presence or not of the conditioned medium of LSECs cultured with the releasate of activated platelets or in the presence or not of the conditioned medium of HSCs cultured with the conditioned medium of LSECs exposed to activated platelets, and we assessed hepatocyte proliferation in these experimental conditions. We confirmed that platelet releasate, LSECs, or the conditioned medium of LSECs cultured with activated platelets, did not increase hepatocyte proliferation, whereas the addition of HSCs to that multicellular cascade led to hepatocyte proliferation. This proliferation was the highest when HSCs were previously exposed to the conditioned medium of LSECs cultured with the releasate of activated platelets (Figure 2E).

Therefore, we conclude that IL-6 released from LSECs upon contact with platelets induces HGF secretion from HSCs, which leads to hepatocyte proliferation.

### 3.3. Platelet Activation and Release of Platelet Granule Content Are Necessary to Induce IL-6 Secretion by LSEC

Thereafter, we aimed at determining the precise mechanism of the above-described multicellular cascade leading to hepatocyte proliferation.

**Figure 3:**
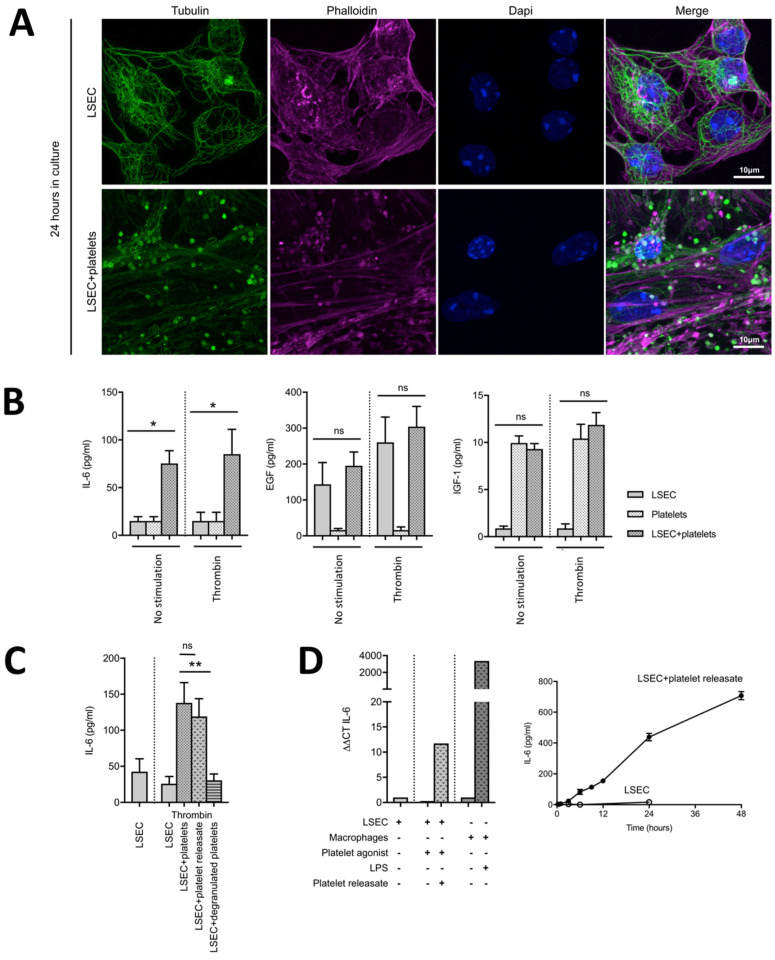
The releasate of activated platelets is necessary to efficiently induce IL-6 release from LSECs. (**A**) LSECs alone and LSECs co-cultured with platelets for 24 h were stained with antibodies against tubulin (green) and phalloidin (magenta, to stain the actin cytoskeleton). Nuclei are stained with dapi (blue). Scale bars, 10 μm. (**B**) Concentrations of IL-6, EGF and IGF-1 at 24 h in the culture medium of LSECs with 16 million platelets without stimulation or with pre-activation using thrombin. N = 4, * *p* < 0.05. (**C**) Secretion of IL-6 at 24 h of culture of LSECs in the presence of either 16 million activated platelets (platelets), their soluble fraction (platelet releasates) or their membrane fraction (degranulated platelets). Platelets and their fractions were obtained after activation by thrombin. N = 8, ** *p* < 0.001. (**D**) Left panel: IL-6 mRNA expression in LSECs cultured for 24 h in the presence or not of activated platelet releasate, platelet agonist, macrophages, and/or LPS. N = 2. Right panel: Kinetics of IL-6 secretion from LSECs alone or LSECs with activated platelet releasate. N = 2.

Resting platelets are characterized by a peripheral ring of microtubules, whereas activated platelets show a more contracted cytoskeleton when aggregated, or dispersed microtubules when spread on a surface [24]. Using immunofluorescence for tubulin, we showed that platelets exhibited a contracted microtubule cytoskeleton when cultured without (Figure S4) or with LSECs for 24 h (Figure S4, Figure 3A), which suggests their activation.

To determine whether platelet activation could increase the effect of platelets on LSECs, platelets were pre-activated by a strong agonist (thrombin [17]) before being cultured with LSECs. We found IL-6 secretion not to be significantly increased when using thrombin-activated when compared to resting platelets (Figure 3B and Figure S4). Releases of EGF and IGF-1 (Figure 3B), as well as other growth factors (Figure S5), after 24 h in culture were not affected by platelet pre-activation. It might be noted, however, that resting platelets already showed activation characteristics in culture (Figure 3A and Figure S4).

Thereafter, we aimed at determining which platelet component was responsible for inducing IL-6 secretion from LSECs. To determine what fraction of platelets induced the observed IL-6 secretion, we cultured LSECs in the presence of activated platelets, releasates from activated platelets (platelet content) or degranulated platelets (activated platelets that have released the content of their granules). Both activated platelets and releasates from activated platelets had similar effects on IL-6 secretion by LSECs. Degranulated platelets had only a minor effect on IL-6 release when compared to whole platelets (Figure 3C). The releasate of activated platelets led to a continuous and cumulative IL-6 secretion by LSECs over time (Figure 3D). Therefore, platelet content is necessary for the platelet-induced IL-6 secretion.

Recent advances in platelet physiology suggested that major platelet granule proteins are differentially stored in platelet α-granules [13] and are distinctly released upon activation of specific PAR [13,14]. Of interest, α-granule release profiles and plasma levels of VEGF and thrombospondin-1 were described to predict liver dysfunction after partial hepatectomy in humans [25]. Moreover, in mice, blocking platelet PAR4 using TcY-NH2 was reported to protect the liver against ischemia-reperfusion injury without impairing liver regeneration [26]. As we observed different IL-6 levels in LSEC cultures after incubation with various platelet activators, we investigated whether selective release of molecules from platelets might occur by stimulation with different agonists. Therefore, we activated mouse platelets using a PAR4 agonist—AY-NH2 (supposed to induce the release of anti-angiogenic factors)—or using thrombin in the presence of a PAR4 antagonist—TcY-NH2—to mimic PAR1 activation (supposed to induce the release of pro-angiogenic factors). However, the LSEC-mediated IL-6 secretion was not significantly different after activation of platelets to release a specific subset of α-granules (Figure S6).

**Figure 4:**
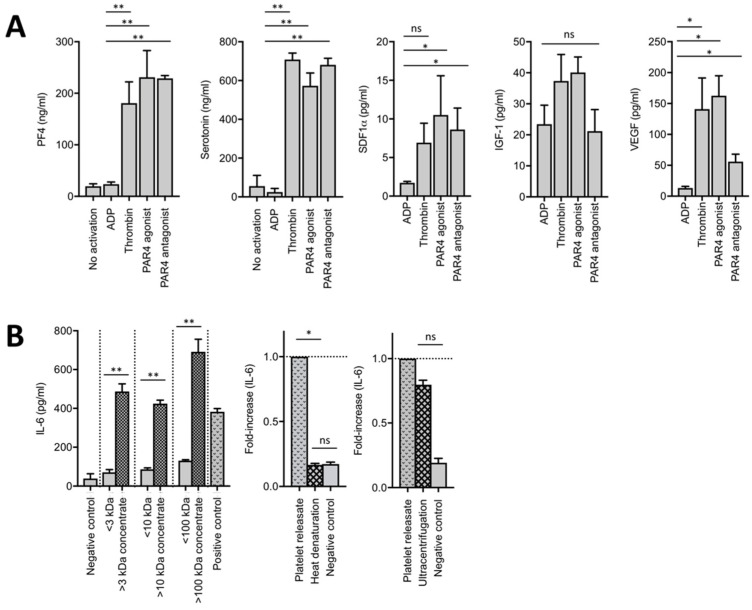
The effect of platelets is mediated through a > 100 kDa protein contained in platelets. (**A**) Concentrations of PF4, serotonin, SDF-1α, IGF-1, and VEGF in releasates of platelets activated either by adenosine diphosphate—ADP, thrombin, a PAR4 agonist, or a PAR4 antagonist associated with thrombin. N = 2–5 (depending on experimental group), * *p* < 0.05, ** *p* < 0.001. (**B**) Left panel: IL-6 secretion at 24 h of culture of LSECs with fractions of the platelet releasates obtained by exclusion after concentration using Amicon centrifugation devices. Middle panel: Ratio of IL-6 secretion from LSECs after heat denaturation of the releasate of activated platelets. N = 3. Right panel: Ratio of IL-6 secretion from LSECs after ultracentrifugation of the releasate of activated platelets. N = 3.

Then, we screened releasates of variously activated platelets for factors known to modulate endothelium regulation. Semi-quantitative analyses using membrane-based antibody arrays identified osteopontin, angiopoietin-1, stromal cell-derived factor 1α: SDF1α, PF4, matrix metalloproteinase-3 (MMP-3), and MMP-8 in the releasates of activated platelets (Figure S7). Then, we measured markers of α- and dense granules releases (PF4 and serotonin, respectively) and molecules with known angiogenic effect (SDF1α, IGF-1, VEGF) in platelet releasates. Both semi-quantitative and quantitative analyses showed that the release profiles of platelet agonists were rather similar in terms of growth factors, with the weak agonist ADP leading to a globally lower release of these molecules than other platelet agonists (Figure 4A and Figure S7).

Therefore, platelet activation and release of content are required for the platelet-induced IL-6 secretion from LSECs. Modulation of the platelet secretome is ineffective in modulating the observed effect using mouse cells.

### 3.4. The Active Component of Platelets Inducing IL-6 Secretion from LSECs Is a > 100 kDa Protein and Is Independent of Platelet Microparticles

In order to narrow down the nature of the specific activity able to induce the observed IL-6 secretion by LSECs, we characterized releasates from activated platelets by subjecting them to different biochemical treatments. Using molecular weight size-exclusion filtration, we determined that the active compound in releasate of activated platelets should have a molecular weight > 100 kDa (Figure 4B). Further, heat denaturation of the platelet releasate completely abrogated its effect on IL-6 secretion by LSECs (Figure 4B, middle panel), suggesting that the active fraction of platelets is of protein nature.

Of interest, Wijten et al. identified by mass spectroscopy that 124 platelet proteins, including seven growth factors, were released after platelet activation [27]. Moreover, we identified SDF-1α and VEGF in the releasates of activated platelets (Figure 4A). Several factors, such as sphingosine-1-phosphate, angiopoietin-1, SDF-1α, VEGF, extracellular matrix protein-1, thrombospondin-1, and platelet-derived growth factor (PDGF) were assessed for their effects on IL-6 secretion from LSECs. However, these factors did not induce IL-6 secretion from LSECs (not shown). Taken together, these results suggest that the active molecule released by platelets and able to induce IL-6 secretion by LSECs may be a protein with a molecular weight above 100 kDa or, alternatively, a smaller molecule associated with a co-factor of a molecular weight > 100 kDa.

Partial hepatectomy was described to generate platelet microparticles [28], < 1.5 µm particles derived from activated platelets [29]. Further, platelet microparticles generated by shear stress were found to induce IL-6 secretion by endothelial cells [30]. Moreover, it has been recently proposed that microRNA-containing microparticles can be delivered to endothelial cells through internalization, resulting in the regulation of gene expression in endothelial cells [31]. Therefore, we hypothesized that platelet microparticles might be involved in liver regeneration [11]. To determine whether such interactions between platelet microparticles and LSECs participate in the observed platelet-mediated IL-6 secretion, we cultured LSECs in the presence of releasates of activated platelets, which underwent ultracentrifugation to remove all extracellular vesicles including microparticles. However, ultracentrifugation of platelet releasates did not abolish the platelet-induced IL-6 secretion by LSECs, suggesting that platelet microparticles do not play a role in IL-6 secretion from LSECs (Figure 4B, right panel).

### 3.5. Adhesion of Platelets to Liver Sinusoids Correlates with Increased Levels of IL-6 after Partial Hepatectomy

The increased IL-6 secretion by LSECs upon platelet interaction with LSECs in vitro led us to investigate the occurrence and physiological impact of interactions between platelets and LSECs after partial hepatectomy in vivo.

**Figure 5:**
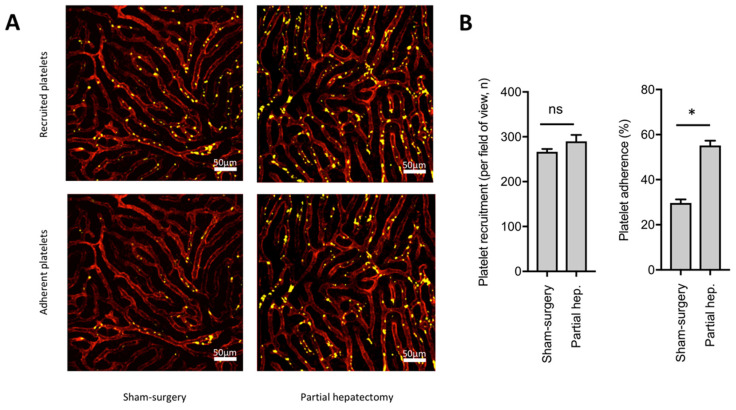
Partial hepatectomy leads to platelets to adhere to LSECs in the liver sinusoids. (**A**) Intravital confocal microscopy was performed in sham-operated (left panels) and partially hepatectomized (right panels) mice. Platelets are shown in yellow and LSECs in red. Upper panels: All platelets present in sinusoids—i.e., recruited platelets in 1 field of view (FOV). Lower panels: Platelets adherent to LSECs—i.e., platelets immobilized ≥ 30 s in sinusoids. (**B**) Quantification of platelet recruitment (left panel) and adherence (right panel) in sinusoids. N = 4, * *p* < 0.05.

First, to determine whether platelets interact with LSECs in liver sinusoids following partial hepatectomy in vivo, mice were injected with PE-conjugated CD49b and AF488-conjugated CD146 antibodies to label platelets and LSECs, respectively. The PE-conjugated CD49b antibody efficiently labeled platelets in vivo, as shown by later in vitro CD41 counterstaining [32] of platelet-rich plasma obtained from those mice (Figure S8). This labeling was still present 24 h after injection (not shown). Thirty-second movies were obtained from the remaining anterior segments of either 60% partially hepatectomized or sham-operated mice using intravital confocal microscopy. These sequences allowed visualization of platelets circulating in the liver sinusoids (Movies S1–S3). Pictures were extracted from these movies to quantify platelet recruitment to the remnant liver (platelets present when image was acquired) and platelet adherence to LSECs (platelets staying immobile > 30 s), in both sham-operated and partially hepatectomized mice (Figure 5). Platelet recruitment to the liver was found to be increased following partial hepatectomy when compared to sham-surgery, but did not reach significance, whereas the proportion of adherent platelets was significantly higher following partial hepatectomy when compared to controls (Figure 5B).

**Figure 6:**
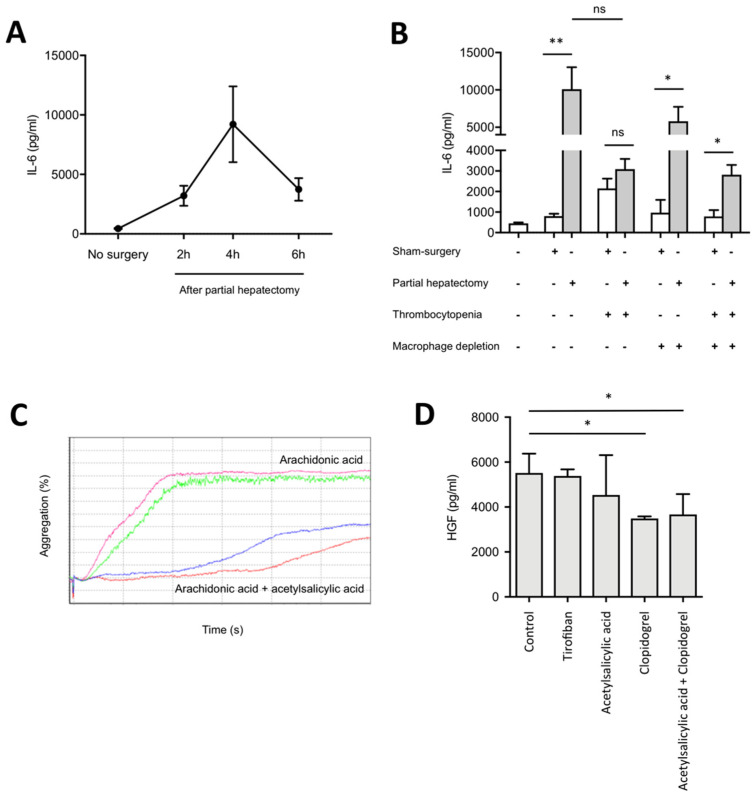
Platelets modulate release of IL-6 and HGF in vivo. (**A**) IL-6 levels in mouse serum after partial hepatectomy. (**B**) Effects of thrombocytopenia on IL-6 levels in mouse serum at 4h following partial hepatectomy in mice with or without macrophage depletion. N = 3–17. (**C**) Aggregation curves of washed platelets with platelet agonist with/without anti-aggregant drug. One mouse per curve. (**D**) Effects of anti-aggregant drugs on HGF levels in mouse serum at 48 h following partial hepatectomy. N = 3–4. * *p* < 0.05, ** *p* < 0.001.

Second, to determine whether platelets are involved in the physiological elevation of IL-6 necessary for the onset of liver regeneration in vivo, severe thrombocytopenia was induced in mice using a platelet-depleting antibody. The platelet-antibody allowed efficient depletion of platelets in sham-operated and partially hepatectomized mice (Figure S9). Mice were sacrificed at the peak of post-operative IL-6 secretion (at 4 h after partial hepatectomy) (Figure 6A). Mice with thrombocytopenia showed high postoperative mortality (not shown) and surviving mice had decreased serum levels of IL-6 when compared to mice with physiological platelet counts, but the result did not reach significance (Figure 6B). In contrast, depletion of macrophages resulted only in a slight reduction of IL-6 levels in hepatectomized mice when compared to sham-operated mice, confirming our in vitro findings that macrophages are not the only source of IL-6 early after partial hepatectomy. Of note, after depletion of both platelets and macrophages, a similar decrease of IL-6 levels was observed as in platelet-depleted mice (Figure 6B).

Third, to determine whether inhibition of platelet function could alter HGF secretion after partial hepatectomy, we determined the optimal concentrations of anti-aggregant drugs allowing inhibiting platelet aggregation (Figure 6C). Then, mice were treated with either acetylsalicylic acid, clopidogrel, acetylsalicylic acid+clopidogrel, or tirofiban before undergoing partial hepatectomy. HGF was determined at 48 h after surgery. Mice treated with clopidogrel, alone or in combination with acetylsalicylic acid had significantly lower blood concentration of HGF after partial hepatectomy than mice injected with saline solution (control) (Figure 6D).

These results show that platelets make contact with LSECs after partial hepatectomy and emphasize the important role of platelets for in vivo early IL-6 elevation and late HGF increase in blood of hepatectomized mice.

## 4. Discussion

Unravelling the trigger of liver regeneration is of crucial interest for clinical practice, in order to develop treatments allowing stimulating recovery of the liver volume in patients suffering from critical alteration of liver functions.

Platelets were shown to contribute to hepatocyte proliferation in vitro and in vivo in rodents [3]. Further, thrombocytopenia was identified as a predictor of post-hepatectomy liver failure in patients after partial hepatectomy [5], and platelet count was reported to correlate with liver volume increase after partial hepatectomy [6]. However, the mechanism by which platelets stimulate hepatocyte proliferation has unfortunately remained unknown.

It is currently admitted that resident macrophages, the Kupffer cells, are one of the most relevant sources for early cytokine release after partial hepatectomy, notably IL-6 [33]. IL-6 and EGF are key to liver regeneration [23,34], and mice defective in IL-6 were reported to develop post-hepatectomy liver failure [35]. However, in this study, we demonstrated that highly pure primary LSECs and also LSEC preparations devoid of macrophages secreted IL-6 and EGF. LSECs did not release HGF, in opposition to results reported by other studies [15]. Further, we observed that freshly isolated syngeneic platelets have a strong impact on IL-6 secretion by LSECs. In fact, the amount of IL-6 secretion was directly correlated to the number of added platelets. Resting platelets, added to LSEC cultures at a concentration of 100,000 platelets/µL, significantly increased IL-6 release by approximately 40-fold when compared to LSECs alone. Our results, using primary differentiated LSECs, underpin the findings of Kawazaki et al. and Nowatari et al., who reported that platelets induce IL-6 release from immortalized human LSECs in vitro [36,37]. Moreover, we showed for the first time that decreased platelet concentrations in vivo, which are already known to correlate with impaired regeneration [38,39], correlated with decreased IL-6 concentrations shortly after partial hepatectomy. Further, depletion of Kupffer cells alone was not sufficient to abrogate IL-6 secretion, confirming that macrophages are not the only source of circulating IL-6 after partial hepatectomy.

These results support the observations of numerous studies summarized in our recent systematic review and meta-analysis, demonstrating that thrombocytopenia constitutes a risk factor for post-hepatectomy liver failure in humans, independently of the presence of liver cirrhosis (which might constitute a confounding factor for observing an effect of thrombocytopenia) [5].

However, we did not observe a direct proliferative effect of platelets on hepatocytes, which is in contrast with the study of Matsuo et al. [40]. Indeed, IL-6 is a known inducer of liver regeneration [23] and triggers hepatocytes from the G0/G1 phase transition after hepatectomy [41]. Moreover, we did not observe an enhanced proliferation of isolated primary hepatocytes exposed to recombinant IL-6 or conditioned medium of LSECs exposed to platelets (containing IL-6). Instead, in our hands, hepatocytes proliferated upon the action of recombinant HGF. Other studies indicated that LSECs release HGF [15,42], but we did not identify HGF release from our primary LSECs when screening the LSEC secretome. Further, regarding the study of Hu et al. [15], we note that LSECs were isolated by magnetic immunosorting using the CD146 surface antigen, which was also considered to be a marker for HSCs [43], leaving undetermined whether LSECs secrete HGF or not, or if the observed results are the product of HSCs.

In our study, we showed that primary HSCs exposed to recombinant IL-6 or to conditioned medium of LSECs (exposed to platelet releasate) secreted HGF, and this was proportional to the concentration of IL-6 contained in the conditioned medium. Further, recombinant HGF, as well as the conditioned medium of HSCs cultured with the conditioned medium of LSECs exposed to platelet releasate (containing, respectively, HGF, and IL-6), stimulated hepatocyte to proliferate. We note that the moderate increase of HGF secretion by HSCs stimulated with recombinant IL-6 indicates that other co-factors released by LSECs are likely to be involved.

Therefore, we conclude that hepatocyte proliferation is not stimulated by platelets or LSECs alone, nor by a combination of both, but rather by a complex multicellular cascade involving platelets, LSECs, and HSCs, ultimately leading to HGF secretion and hepatocyte proliferation.

Thereafter, we aimed at identifying the initial trigger of that process. Of note, evidence suggests that platelets release their content following partial hepatectomy in vivo [44,45] and that the platelet content directly affects hepatocyte proliferation in vitro and in vivo [39,40,46]. Therefore, we aimed at determining whether platelet activation, i.e., platelet granule release, potentiates platelet-induced IL-6 secretion by LSECs. First, using platelet releasates versus degranulated platelets, we found that induction of IL-6 secretion was dependent on platelet releasates rather than on degranulated platelets. This revealed that the platelet secretome is sufficient to induce IL-6 secretion and further suggests that the interaction of platelets and LSECs is mainly required for platelet activation and granule release. Furthermore, we analyzed platelet releasates to determine the mechanism of action on LSECs. We excluded hypothetical contribution of platelet microparticles [11,28] to the observed induction of IL-6 secretion by LSECs and suggested that the active factor(s) present in platelet releasates has a molecular weight above 100 kDa and is heat-sensitive. Considering these results, the participation in that process of serotonin [39,47], as well as of the recombinant molecules we have tested (sphingosine-1-phosphate, angiopoietin-1, SDF-1α, VEGF, extracellular matrix protein-1, thrombospondin-1, and PDGF), is unlikely.

After demonstrating the effects of contact between platelets and LSECs, we aimed at determining whether platelets interact with LSECs in liver sinusoids following partial hepatectomy in vivo. Using intravital confocal microscopy associated with a method for in vivo endogenous platelet labeling, we observed increased adherence of platelets to sinusoids early after partial hepatectomy. We note that, using exogenous transfused labeled-platelets, Matsuo et al. showed those platelets to be recruited to liver sinusoids following partial hepatectomy in rats [48]. Platelets were also described to adhere to endothelial cells early after thermally-induced focal liver injury and to remain adherent for 4–8 h [49], suggesting that platelet adherence to sinusoids is not specifically induced after loss of liver tissue by resection. Herein, we showed, using intravital confocal microscopy associated with a method for in vivo endogenous platelet labeling, that partial hepatectomy leads to the early adherence of platelets to LSECs. The adhesion of platelets to sinusoids and its correlation with increased IL-6 levels in mice during early liver regeneration suggest that local IL-6-secretion by LSECs is necessary for triggering liver regeneration. Specific knock-out of the IL-6 gene in LSECs could help to determine the impact of IL-6 secretion from LSECs in liver regeneration. In our hands, death of thrombocytopenic mice after partial hepatectomy did not allow to efficiently analyze liver regeneration in vivo. We overcame that limitation by showing that anti-aggregant drug clopidogrel significantly inhibited release of HGF after partial hepatectomy.

To conclude, our findings provide new insights regarding the implication of platelets in liver regeneration. Platelets may not only have a direct mitogenic effect on hepatocytes, as previously shown in the literature, but also act indirectly via interactions with non-parenchymal cells [3,16]. Our results demonstrate that platelets adhere to LSECs following partial hepatectomy and suggest that this contact stimulates LSECs to release IL-6, which, in turn, stimulates secretion of HGF by HSCs. Therefore, our study suggests that the beneficial effect of platelets on the regenerative process [3,5] relies on early interactions with LSECs and then HSCs and that activated platelets constitute the trigger of liver regeneration. Future analytical studies are needed to identify the molecular actor of this phenomenon among the 124 identified proteins of the platelet proteome [27]. The identification of the molecule(s) implicated will be a scientific breakthrough and will open new perspectives for patients suffering from acute liver failure, post-hepatectomy liver failure, or small-for-size syndrome after liver transplantation, but also allows performing hepatectomies of a greater extent in cancer patients.

## Supplementary Materials

The following are available online at https://www.mdpi.com/2073-4409/9/5/1243/s1, Figure S1: Protocols for platelet preparations, Figure S2: Growth factor release from LSECs after incubation with increasing numbers of resting, Figure S3: IL-6 release by resident macrophages is not modulated by platelets, Figure S4: Respective proportions of resting and activated platelets in culture, Figure S5: Growth factor release from LSECs after incubation with increasing numbers of thrombin-activated platelets, Figure S6: PAR4 does not modulate the effects of platelets on IL-6 secretion, Figure S7: Screening of growth factors in platelet releasates, Figure S8: Platelet staining for intravital confocal microscopy, Figure S9: Platelet counts in mice injected with platelet-depleting antibodies, Figure S10: Quantification of recruited and adherent platelets using intravital confocal microscopy. Video S1: Streaming movie of a sham-operated mouse acquired using continuous 561 nm singlewavelength stimulation and merged with a 488 nm non-simultaneously acquired movie of the same field of view. Video S2: Movie of a sham-operated mouse generated by merging sequential acquisitions of the 561 nm and 488 nm channels 15 min after surgery. Video S3: Movie of a 60% partially hepatectomized mouse generated by merging sequential acquisitions of the 561 nm and 488 nm channels 15 min after surgery.
