# Supplementary material for: Platelet Interactions with Liver Sinusoidal Endothelial Cells and Hepatic Stellate Cells Lead to Hepatocyte Proliferation
Source: Cells. 2020 May 18;9(5):1243. doi: 10.3390/cells9051243 (PMC7290338; doi:10.3390/cells9051243)
Supplement: Supplementary file 1 [file cells-09-01243-s001.zip › cells-797594-supplementary.pdf]

cells

Article

Platelet Interactions with Liver Sinusoidal Endothelial Cells and Hepatic Stellate Cells Lead to Hepatocyte Proliferation

Supplementary Materials

1. Materials and Methods

Materials

CD11b microbeads for mice, LS columns, QuadraMACS separator, and MACS MultiStand platform were purchased from Miltenyi Biotec (Bergisch Gladbach, Germany). ELISA kits for mouse HGF, IL-6, EGF, VEGF, IGF-1, TGF-β, and SDF-1α were acquired from RayBiotech (Norcross, USA). ELISA kit for mouse/rat HGF was purchased from R&D Systems (Minneapolis, USA). ELISA kits for mouse IL-6, PF4, and serotonin were from Abcam (Cambridge, United Kingdom). Mouse Angiogenesis Array Kit (Proteome Prolifer) was from R&D Systems (Minneapolis, USA). Amiconultra 0.5 centrifugal filter device was purchased from Merck Millipore (Billerica, USA). Oil-Red-O solution was obtained from Sigma-Aldrich (Buchs, Switzerland).

Reagents

Type 4 collagenase 320 units/mg was provided by Worthington Biochemical Corporation (Lakewood, USA). Type 1 DNase 2000 units/mg was purchased from Roche Diagnostics (Risch, Switzerland). OptiPrep density gradient medium was obtained from Axis-Shield (Oslo, Norway). Nycodenz density gradient medium was purchased from Axon lab AG (Le Mont-sur-Lausanne, Switzerland). Percoll density gradient medium was provided by GE healthcare (Little Chalfont, United Kingdom). Endothelial cell medium for animals was obtained from Cell Biologics (Chicago, USA). Arginine-free, glutamine-free William's E medium was a courtesy of Prof. D. Stroka (University of Bern, Switzerland). Hydrocortisone, L-ornithine, and L-glutamine were obtained from Sigma-Aldrich (Buchs, Switzerland). PAR4 agonist AY-NH2 and PAR4 antagonist TcY-NH2 were purchased from Tocris Bioscience (Bristol, United Kingdom). Recombinant IL-6 was obtained from Biolegend (San Diego, USA). Recombinant HGF, TGF-β, SDF1, VEGF, and PDGF were purchased from PreproTech (Rocky Hill, USA). Recombinant angiopoietin-1 and ECM1 were purchased from R&D systems (Minneapolis, USA). Sphingosine-1-posphate was obtained from Sigma-Aldrich (Buchs, Switzerland). All other reagents were of analytical grade.

Antibodies and Acetylated Low-Density Lipoprotein

Purified anti-mouse CD16/32 (93), APC anti-mouse F4/80 (BM8), PE anti mouse CD49b (Hmα2), AF488 anti-mouse CD146 (ME-9F1), and AF488 anti-mouse CD41 (MWReg30) were purchased from Biolegend (San Diego, USA). AF488 anti-mouse stabilin-2 (#34-2) was from MBL International (Woburn, USA). Unconjugated anti-mouse integrin β3 (Luc.A5), anti-mouse CD42b (GPIbα) platelet-depleting antibody, and rat IgG isotype control were obtained from Emfret Analytics (Würzburg, Germany). Unconjugated anti-mouse tubulin (B-5-1-2) was purchased from Sigma-Aldrich (Buchs, Switzerland). AF555 anti-rabbit was obtained from Invitrogen (Carlsbad, USA). Unconjugated anti-mouse α-SMA was a courtesy of Dr. S. Clément (University of Geneva, Switzerland). Click-it EdU assay AF488 with Hoechst was purchased from Thermo Fisher Scientific (Waltham, USA). Antibodies are reported in the Supplementary CTAT table.

cells

Cell isolation Procedures

Digestion Step

According to the protocol previously published [1], mice were anesthetized and exsanguinated by cardiac puncture. The liver was cannulated through the supra-hepatic portion of the inferior vena cava and perfused for 5 min using wash solution (calcium-free HBSS, penicillinstreptomycin, heparin, glucose, HEPES, EGTA). The portal vein was immediately sectioned at the beginning of perfusion. Then, the liver was perfused with collagenase solution (IMDM, 0.1% collagenase, 0.008% DNase) under a warming infrared light for 5 min. The liver was excised, placed on a Petri dish filled with post-digestion solution (DMEM, 5% FCS, penicillin-streptomycin), and mechanically disrupted. Digested liver tissue was filtered through a 70 µm cell strainer.

LSEC Isolation

Digested liver tissue was centrifuged at 68 xg for 5 min to pellet parenchymal cells. To collect non-parenchymal cells, the supernatant was centrifuged at 600 xg for 10 min. The pelleted cells were suspended in an 8.2/17.6% Optiprep gradient and centrifuged at 1400 xg for 30 min, with acceleration and deceleration set to minimal values. Cells located at the interface between the two density cushions were collected, suspended in MACS buffer, and centrifuged at 780 xg for 10 min. The pelleted cells were incubated with 60 µL anti-CD11b microbeads to label contaminating macrophages and passed successively through 2 MACS columns placed on a MACS separator. Columns were rinsed once with 2 mL MACS buffer. The flow-through containing the CD11b-negative cells was centrifuged at 600 xg for 10 min. Cell viability was evaluated by counting dead cells stained by 0.4% trypan blue solution.

To further plate LSECs with requested densities, proportion of LSECs in the CD11b-negative fraction was determined. 100,000 cells were collected and centrifuged at 600 xg for 5 min. Sampled cells were suspended in flow cytometry buffer, incubated with Fc-receptor blocking CD16/32, then incubated with APC-conjugated F4/80 and AF488-conjugated stabilin-2, washed, and fixed. Cells were acquired using an Accuri C6 flow cytometer (BD Biosciences, Bedford, USA) and analyzed on Flowjo version 10 (Tree Star Inc., Ashland, USA). After gating for the cell population, expressions of stabilin-2 and F4/80 were analyzed. The yield of LSECs from one liver was determined by multiplying the proportion of stabilin-2+ F4/80- cells (LSECs) obtained by the flow cytometry analysis with the total number of cells counted using a hemocytometer.

For experiments, cells were then suspended in endothelial cell medium and seeded at a density of 100,000 LSECs per well on non-coated 96-well plates. The plates were centrifuged at 160 xg for 2 min and cells were incubated at 37 °C for 2 h to select for adherent LSECs (long-term selective adherence step). The culture medium containing non-adherent cells was then discarded and adherent cells were rinsed thrice with DPBS. Endothelial cell medium was added, and cells were kept at 37 °C for further experimental procedures.

Purity of cultured LSECs was determined for each isolation procedure. A sample of cultured cells was trypsinized, centrifuged at 600 xg for 10 min and prepared for flow cytometry using stabilin-2 and F4/80 staining. Expressions of stabilin-2 and F4/80 were analyzed. Purity was expressed as the ratio of stabilin-2+ F4/80- cells on the total number of gated cells.

For immunofluorescence, cells were fixed using isotonic formalin for 15 min at RT, then permeabilized with PBS, 0.2% Triton-X-100 for 15 min and incubated in blocking buffer (PBS/3% BSA/10% goat serum) for 1h before incubation with primary antibodies (anti-stabilin-2, MBL, D317-A48; anti-tubulin, Sigma, T5168), followed by incubation with secondary antibodies and/or phalloidin and DAPI (4′,6-Diamidine-2′-phenylindole dihydrochloride).

Resident Macrophage Isolation

During the magnetic sorting step of LSEC isolation, columns were removed from the magnetic field, and macrophages were harvested according to manufacturer's instructions. Cells were centrifuged at 600 xg for 10 min. The proportion of macrophages was determined using flow cytometry for F4/80. Cells were suspended in endothelial cell medium and seeded at a density of 75,000 macrophages per well on a non-coated 96-well plate. Plates were further processed as for LSECs.

HSC Isolation

Digested liver tissue was centrifuged at 68 xg for 5 min to pellet parenchymal cells. The supernatant was centrifuged at 610 xg for 10 min at 4 °C. Pelleted cells were suspended in GBSS with NaCl with type I DNase and centrifuged at 610 xg for 10 minutes at 4 °C. Then, cells were suspended into 13% Nycodenz, according to Mederacke et al. [2]. HSCs were retrieved from the interface between the two gradient cushions, washed, suspended into IMDM 10% FCS, and centrifuged at 690 xg for 15 min at 4 °C. Pelleted cells were suspended into IMDM 10% FCS, counted, and plated on 96-well plate at a density of 100,000 cells/well. Culture medium was changed after 12 h. Purity was assessed by autofluorescence of vitamin A droplets, incorporation of Oil-red-O, but also by performing immunofluorescence for α-SMA after 7 days in culture. For immunofluorescence, HSCs were cultivated on 35 mm plastic dishes and microdrop plated. Medium was added 2 h after cell adhesion, and cells were washed after 12 h. After 36 h, medium was changed, and TGF-β was added. Cells were fixed using isotonic formalin for 15 min at RT, then permeabilized with PBS, 0.1% Triton-X-100 for 15 min, and incubated in blocking buffer (PBS/3% BSA/10% goat serum) for 1h before incubation with primary antibodies (anti-mouse α-SMA), followed by incubation with secondary antibodies and/or Hoechst.

Hepatocyte Isolation

Digested liver tissue was centrifuged at 68 xg for 5 min to pellet parenchymal cells. Pelleted cells were suspended into 25 mL post-digestion solution (DMEM, 5% FCS, penicillin-streptomycin) and washed twice. Cells were then suspended in 54% Percoll solution. The pellet was washed twice and centrifuged at 122 xg. Pelleted cells were resuspended in 12.5 mL of post-digestion solution (DMEM, 5% FCS, penicillin-streptomycin). Cells were counted and suspended into Williams' E medium, 10%FCS, penicillin, and streptomycin at a density of 18,000 cells/well in 96-well plate. Medium was changed after 3 h. After 12 h, culture medium was changed for hepatocyte culture medium (arginine-free, glutamine-free William's E, 0.4 mmol/L L-ornithine, 2 mmol/L L-glutamine, 2 g/mL hydrocortisone, and penicillin/streptomycin).

Screening for Growth Factors in the Platelet Releasates

Sixteen million platelets per condition were activated, as previously described, supplemented with 2.5µL/mL PGI2 and centrifuged at 600 xg for 15 min. The supernatant was harvested and analyzed using a proteome profiler array for cytokines and growth factors related to angiogenesis, as well as ELISA assays for platelet factor 4 (PF4, a marker for platelet α-granules release [3]), serotonin (a marker for dense granule release [3]), VEGF [3–5], IGF-1 [6,7], and SDF-1α [3–5,8].

Platelet Releasate Concentration

Platelet releasates were concentrated with 3 and 10 kDa filter, according to the manufacturer's instructions. The filtered and concentrated fractions were recovered and added to primary LSECs in culture.

Protein Denaturation

Platelet releasates were heated at 95 °C for 15 min and added to primary LSECs in culture.

Ultracentrifugation

Ultracentrifugation of platelet releasates was performed with a Beckmann L7-55 Ultracentrifuge (Beckman Coulter, Brea, USA), using a SW-55Ti swinging-bucket rotor at 33,000 revolutions per minute, which corresponds to a relative centrifugal force of about 100,000 xg [9]. Centrifugation was performed for 1 h at 4 °C. The supernatant was collected and added to primary LSECs in culture.

Assays for Growth Factors in the Co-Culture Medium

The co-culture medium of LSECs with platelets, or of HSCs and conditioned medium of LSECs and with platelets, was harvested after 24 h and centrifuged at 600 xg for 2 min. The supernatant was stored at −20 °C. ELISA was performed for growth factors demonstrated to be involved in platelet-mediated hepatocyte proliferation, i.e., hepatocyte growth factor (HGF) [7], IL-6 [10], insulin-like growth factor-1 (IGF-1) [7], vascular endothelial growth factor (VEGF) [10], endothelial growth factor (EGF), and transforming growth factor-β (TGF-β), according to manufacturers' instructions.

Hepatocyte Proliferation Assay

For proliferation experiments with conditioned medium from LSECs exposed to platelets, hepatocytes were cultured for 36 h in complete hepatocyte medium, washed, and then cultured in William's Medium 0.1% FCS for 12 h. Then, hepatocytes were washed and conditioned medium of 100,000 LSECs cultured with 16 million platelets for 24 h, and 1/1000 EdU were added to each well. Hepatocytes were cultured for 24 h, washed, fixed, and processed for EdU immunofluorescence. For experiments with recombinant cytokines and growth factors, hepatocytes were cultured for 12 h in William's medium supplemented with 10% FCS and penicillin/streptomycin. Then, hepatocytes were cultured in arginine-free, glutamine-free William's medium supplemented with L-ornithine (0.4 mmol/L), L-glutamine (2 mmol/L), hydrocortisone (2 $g/ml), and penicillin/streptomycin for 24 h. Then, hepatocytes were cultured for 24 h with various concentrations of IL-6, HGF and transforming growth factor-β (TGF-β) in William's medium in the presence of 1/1000 EdU. Hepatocytes were fixed and processed for EdU immunofluorescence.

Partial Hepatectomy

Partial hepatectomy was performed by removing the left posterior, omental, right middle, and right posterior segments, which corresponds to the removal of 60% of the liver parenchyma [11], using ligatures and sections under visual control with a surgical microscope (M650, Wild, Heerbrugg, Switzerland). Careful hemostasis was applied on the section cuts using a small vessel cauterizer. For experiments using intravital confocal microscopy, removal of the liver lobes was performed using the clip technique [11], allowing to leave the right anterior and left anterior segments untouched. sham surgery consisted in median laparotomy and mobilization of the liver lobes with cotton swabs.

Macrophage Depletion

Macrophage depletion was performed by intra-peritoneal injection of 200 µL of clodronate liposomes (www.clodronateliposomes.org, Haarlem, The Netherlands) 24 h before experimentation.

Intravital Confocal Microscopy

Intravital confocal microscopy for mouse liver was performed according to a method previously described [12] with some modifications. Briefly, mice were anesthetized with ketamine. Four µg PE-conjugated CD49b and 20 µg AF488-conjugated CD146 antibodies were injected into inferior vena cava to in vivo label platelets [12] and LSECs [13], respectively. Mice were either partially

hepatectomized or sham-operated. After 15 min, the right anterior segment was carefully exteriorized on a glass cover slip and kept moist. Liver intravital imaging was performed using a spinning-disk confocal microscope (Cell Observer Z1, Carl Zeiss, Oberkochen, Germany) fitted with 10x/0.3 NA and 20x/0.5 NA inverted objectives and equipped with a Yokogawa confocal scanner unit (Musashino, Japan). Fluorophores AF488 and PE were optimally excited by 488 nm and 561 nm diode lasers, respectively. Pictures were collected with an Evolve EMCCD camera (Photometrics, Tucson, USA) piloted by MetaMorph software (Molecular Devices, Sunnyvale, USA). Six movies from random 200x fields of view were acquired per mouse and analyzed by an operator blinded for groups on ImageJ software (National Institutes of Health, Bethesda, USA) according to a method previously described with modifications [14] (Figure S10). Recruited platelets were defined as the total number of platelets on 1 static field of view. Adherent platelets were defined as the total number of platelets that remained immobile on a 30s sequence [12]. The proportion of adherent platelets was calculated as the ratio of adherent platelets divided by recruited platelets.

Thrombocytopenia

Mice were injected intra-peritoneally with 1 μg/mg polyclonal GPIbα in 150 μL NaCl 0.9% 36 h before surgery, as previously described [15]. Control mice were injected intra-peritoneally with isotype control, when indicated. Platelet counts were determined at sacrifice using an automated hematology analyzer (Sysmex KX-21N, Sysmex Suisse AG, Yverdon-les-Bains, Switzerland).

In vivo Platelet Anti-Agregation

First, we determined minimal concentration of various platelet agonists inducing platelet aggregation. Briefly, blood was collected from C57BL/6 male mice by cardiac puncture into acid-citrate-dextrose solution and centrifuged at 100 xg for 15 min to obtain platelet-rich plasma. Platelet-rich plasma was supplemented with 2.5 μL/Ml PGI2 and centrifuged at 600 xg for 15 min to obtain washed platelets. Washed platelets were counted manually and adjusted to 200,000 platelets/μL using Tyrode's buffer. After 30 minutes, minimal concentrations of arachinodic acid, ADP + fibrinogen or thrombin inducing platelet aggregation were selected by titration using a TA-8V aggregometer (SD Medical, Heillecourt, France). Second, C57BL/6 male mice were injected intraperitoneally 1x/day with various concentrations of anti-aggregant drugs inhibiting different pathways of platelet aggregation (acetylsalicylic acid, clopidogrel, and tirofiban) for 7 days. Blood was then collected by cardiac puncture and processed as previously described to obtain washed platelets. Concentrations of anti-aggregant drugs allowing inhibition of platelet aggregation were determined. Third, C57BL/6 male mice were injected intraperitoneally 1x/day with either 1mg/kg/day acetylsalicylic acid, 1mg/kg/day clopidogrel, or a combination of both for 7 days. Four mice received only one injection of 50 μg/kg tirofiban in the inferior vena cava one minute before partial hepatectomy. Control mice received saline injection for 7 days. On day 7, partial hepatectomy was performed. At 48 hours after surgery, blood was collected as previously described. ELISA assay was performed for HGF.

2. Supplementary Figures

cells

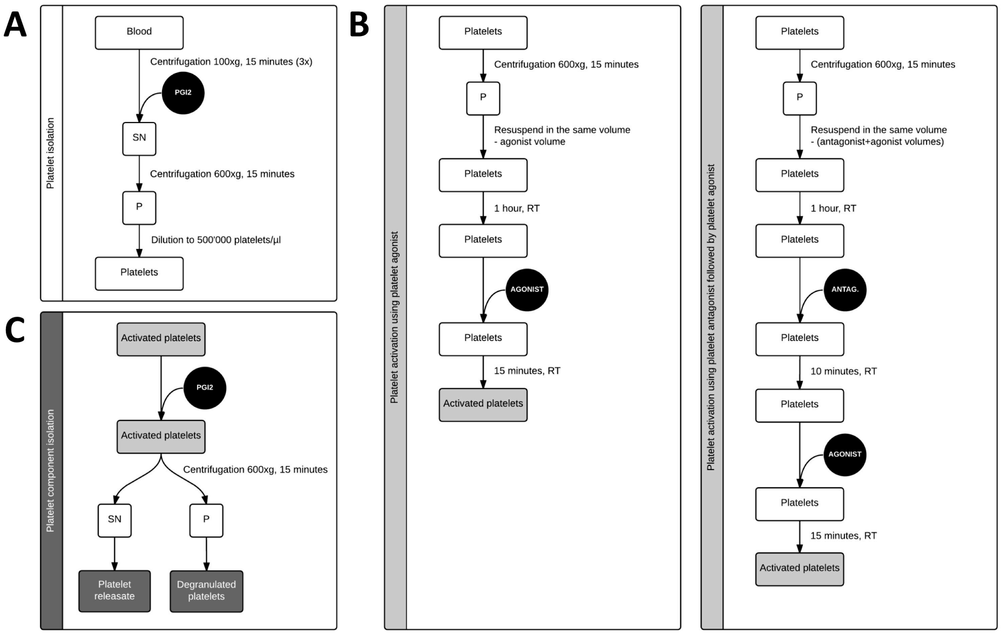

Figure S1. Protocols for platelet preparations. A. Platelet isolation. B. Platelet activation via specific receptors using agonists and antagonists. C. Preparation of platelet fractions (platelet releasates and degranulated platelets).

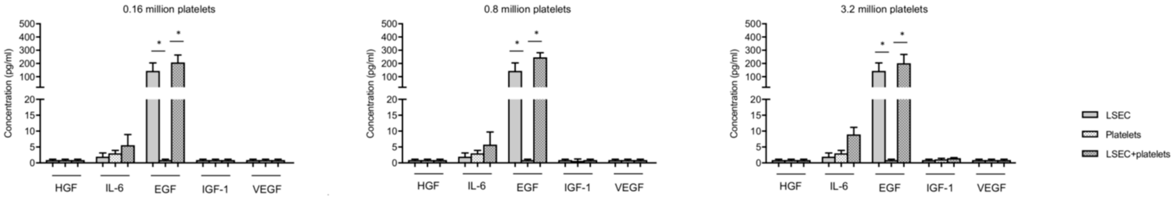

Figure S2. Growth factor release from LSECs after incubation with increasing numbers of resting platelets. Concentrations of growth factors present after 24 h in low-FCS culture medium of LSECs alone or with increasing concentrations of resting platelets. N = 4, * $p < 0.05$.

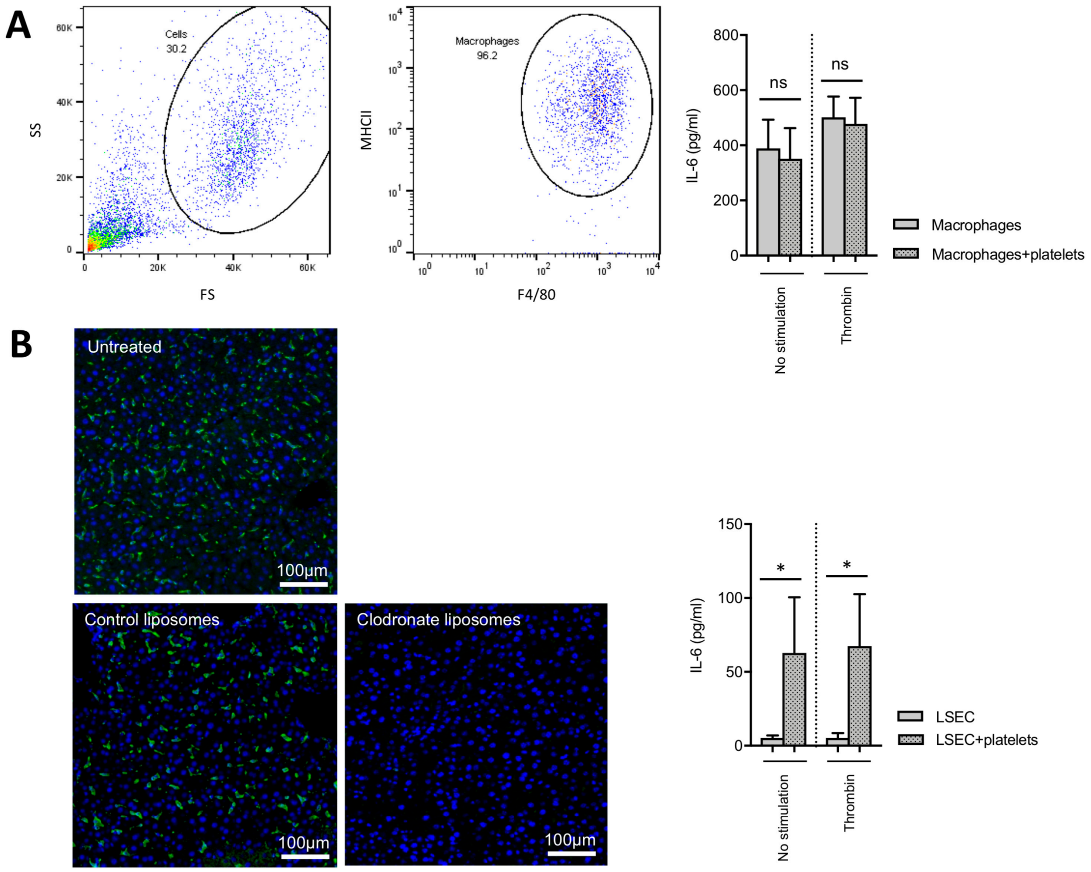

Figure S3. IL-6 release by resident macrophages is not modulated by platelets. A. Dot-plots of flow cytometry analysis of isolated CD11b-positive cells. Left panel: Gating strategy. Middle panel: purity of primary macrophages according to F4/80 and MHCII staining. Right panel: IL-6 release measured in the culture medium of isolated macrophages with or without resting and activated platelets. B. Left panels: Immunofluorescence staining of liver sections using antibody against IBA-1 (green) for macrophages in untreated or clodronate-treated mice; nuclei are stained with dapi (blue). Right panel: IL-6 release measured in the culture medium of LSECs isolated from clodronate-treated (macrophage-depleted) mice with resting and activated platelets. N = 4, * $p < 0.05$.

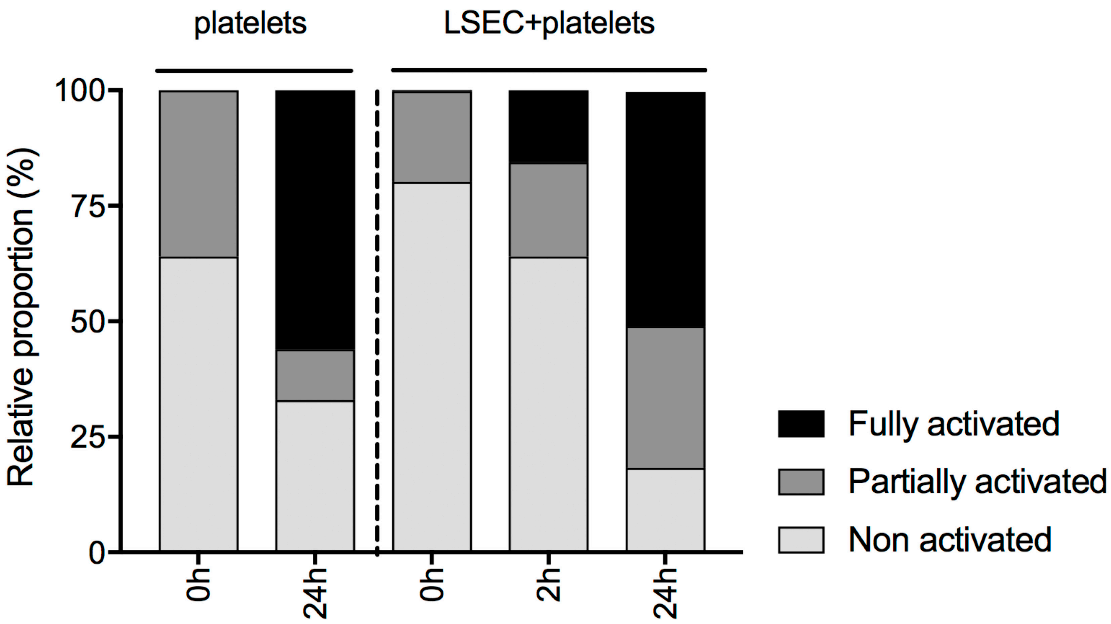

Figure S4. Respective proportions of resting and activated platelets in culture. Platelets were manually counted using a 2xplan-Apochromat 63x/1.40 Oil objective. Partially activated platelets were defined by the presence of a coiled marginal bands. Fully activated platelets featured a micro marginal band. Resting platelets had a regular size and shape marginal bands.

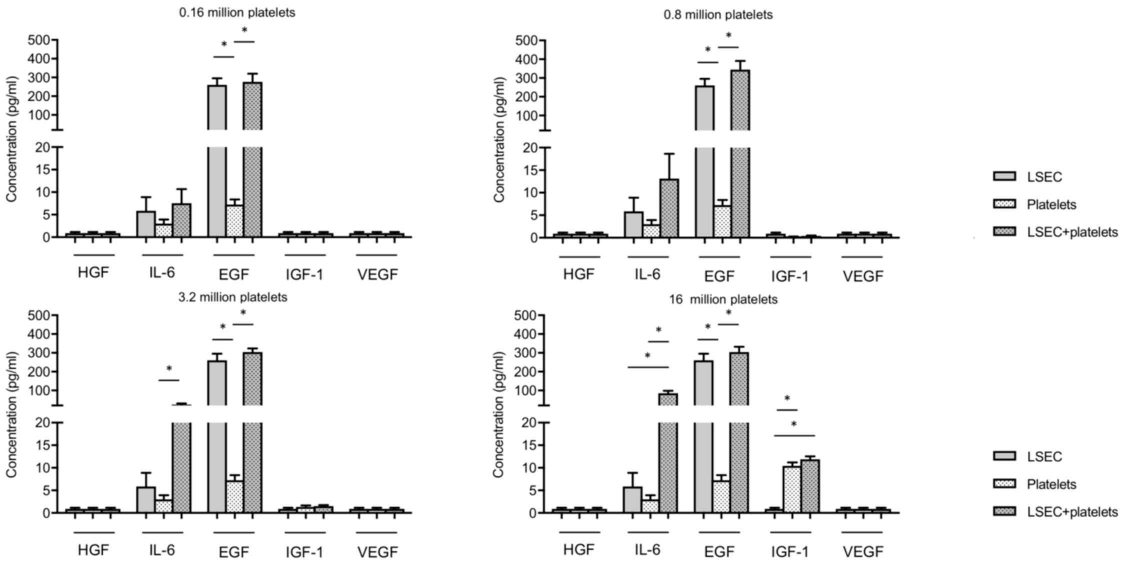

Figure S5. Growth factor release from LSECs after incubation with increasing numbers of thrombin-activated platelets. Concentrations of cytokines and growth factors present after 24 h in low-FCS culture medium of LSECs alone or with increasing concentrations of thrombin-activated platelets. N = 4, * $p < 0.05$.

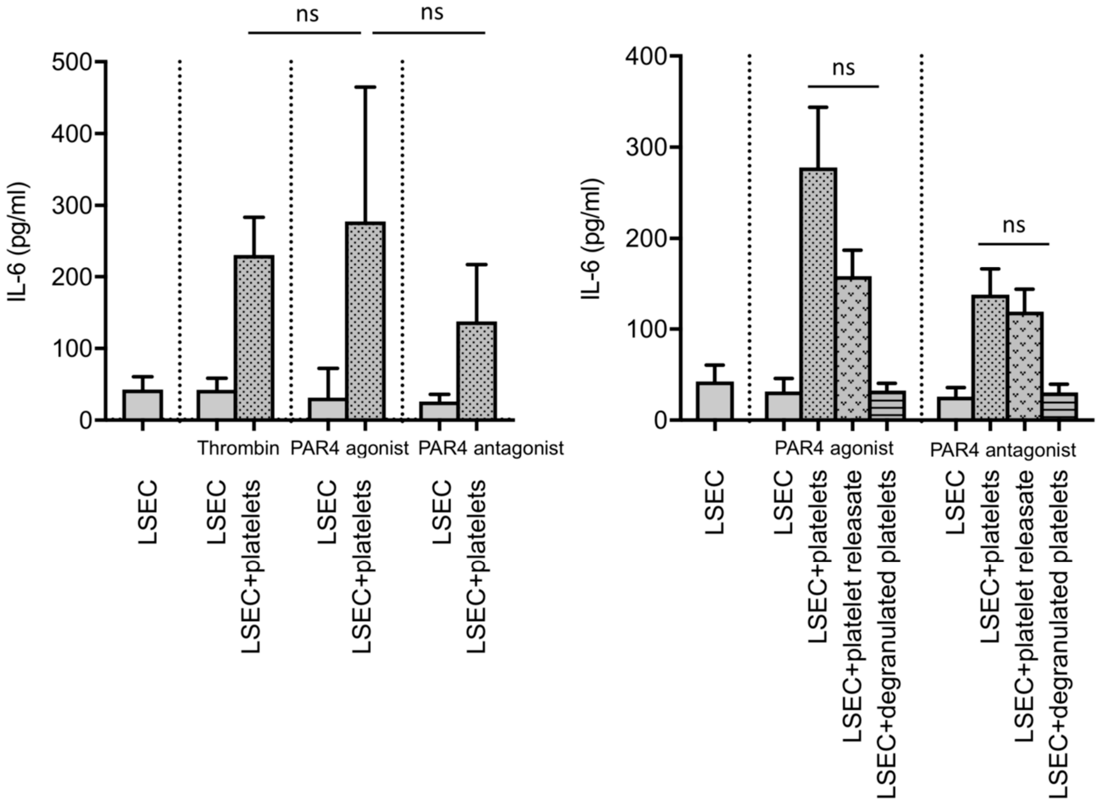

Figure S6. PAR4 does not modulate the effects of platelets on IL-6 secretion. A. Secretion of IL-6 at

24 h of culture in the presence of 16 million activated platelets activated either by thrombin, a PAR4 agonist, or a PAR4 antagonist associated with thrombin. N = 8, * $p < 0.05$, ** $p < 0.001$. B. N = 2. B. Secretion of IL-6 at 24 h of culture in the presence of either 16 million activated platelets (platelets), their soluble fraction (platelet releasates) or their membrane fraction (degranulated platelets). Platelets and their fractions were obtained either after activation by a PAR4 agonist or by a PAR4 antagonist associated with thrombin. N = 8.

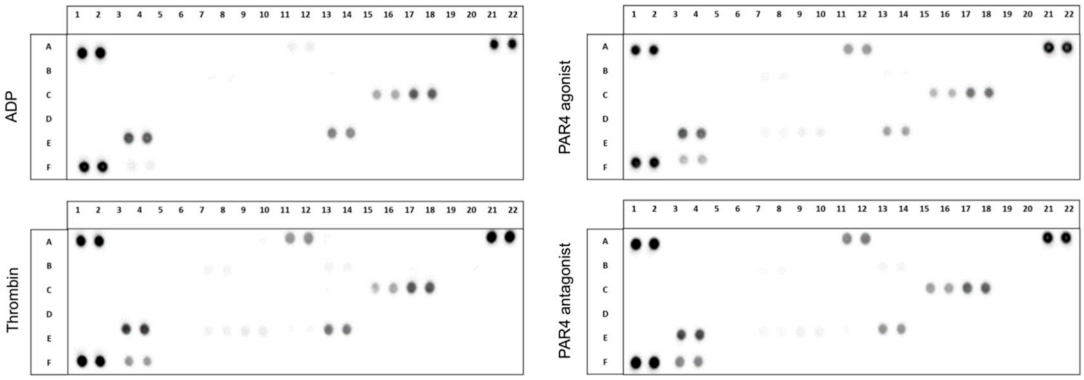

	1	2	3	4	5	6	7	8	9	10	11	12	13	14	15	16	17	18	19	20	21	22
A	Pos	Pos	-	-	ADAMTS 1	ADAMTS 1	Amphiregulin	Amphiregulin	Angiogenin	Angiogenin	Angiopoietin-1	Angiopoietin-1	Angiopoietin-3	Angiopoietin-3	Tissue Factor	Tissue Factor	CXCL16	CXCL16	-	-	Pos	Pos
B	-	-	IGFBP-10	IGFBP-10	DLL4	DLL4	CD26	CD26	EGF	EGF	Endoglin	Endoglin	Endostatin	Endostatin	Endothelin-1	Endothelin-1	FGF-1	FGF-1	FGF-2	FGF-2	-	-
C	-	-	FGF-7	FGF-7	Fractalkine	Fractalkine	GM-CSF	GM-CSF	HB-EGF	HB-EGF	HGF	HGF	IGFBP-1	IGFBP-1	IGFBP-2	IGFBP-2	IGFBP-3	IGFBP-3	IL-1α	IL-1α	IL-1β	IL-1β
D	-	-	IL-10	IL-10	CXCL10	CXCL10	CXCL1	CXCL1	Leptin	Leptin	MCP-1	MCP-1	MIP-1α	MIP-1α	MMp-3	MMP-3	MMP-8	MMP-8	MMP-9	MMP-9	IGFBP-9	IGFBP-9
E	-	-	Osteopontin	Osteopontin	PD-ECGF	PD-ECGF	PDGF-AA	PDGF-AA	PDGF-AB/BB	PDGF-AB/BB	PTX3	PTX3	PF4	PF4	PIGF-2	PIGF-2	Prolactin	Prolactin	Proliferin	Proliferin	-	-
F	Pos	Pos	SDF-1	SDF-1	Serpin E1	Serpin E1	Serpin F1	Serpin F1	TSP-2	TSP-2	TIMP-1	TIMP-1	TIMP-4	TIMP-4	VEGF	VEGF	VEGF-B	VEGF-B	Neg	Neg		

Figure S7. Screening of growth factors in platelet releasates. Semi-quantitative screening and comparison of growth factor levels in platelet releasates activated with different agonists. N = 1.

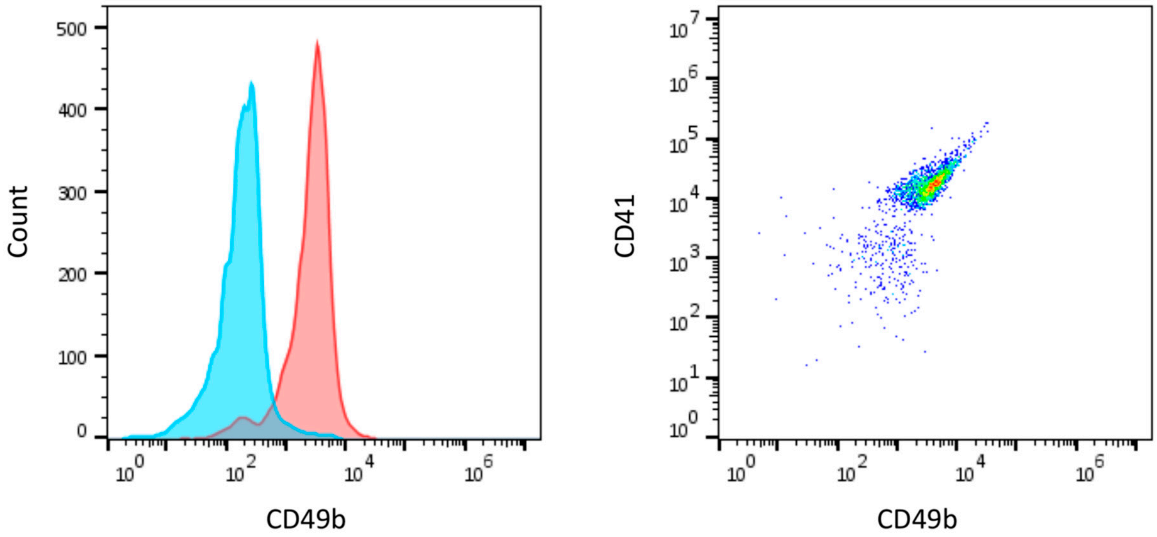

Figure S8. Platelet staining for intravital confocal microscopy. A. Platelet-rich plasma from mice injected in vivo with PE-conjugated anti-CD49b was used to demonstrate specificity of platelet labelling. Left panel: Histogram of stained platelets showing isotype control (blue) and anti-CD49b antibody (red). Right panel: Dot plot of in vivo labelled platelets (PE-conjugated anti-CD49b) counterstained in vitro with AF488-conjugated anti-CD41.

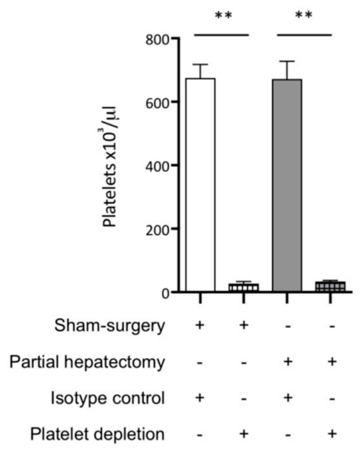

Figure S9. Platelet counts in mice injected with platelet-depleting antibodies. N= 3–17, * $p < 0.05$, ** $p < 0.001$.

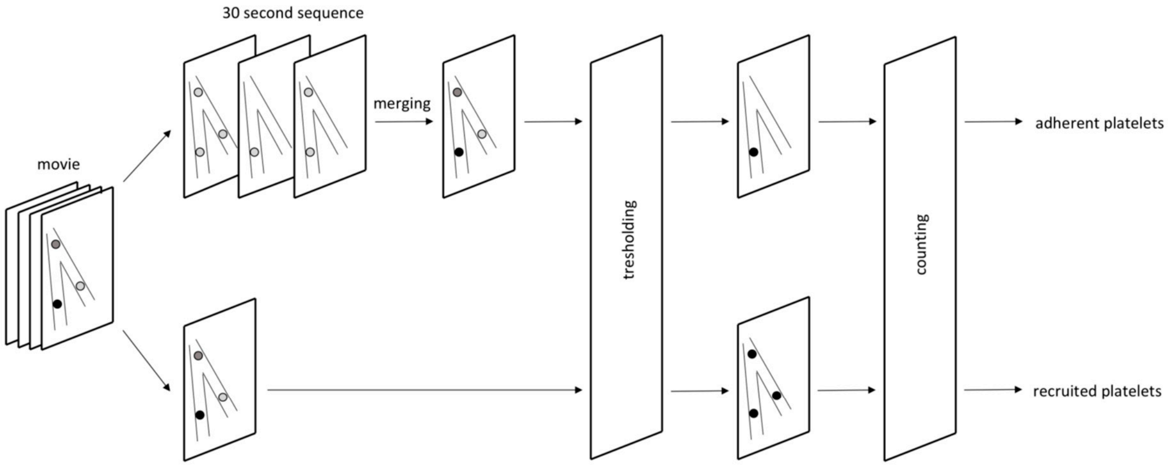

Figure S10. Quantification of recruited and adherent platelets using intravital confocal microscopy.

Pictures acquired from the red channel (platelets) corresponding to a 30 s sequence movie were converted to 8-bit images and anonymized. Automatic brightness and contrast adjustment as well as smoothing pixels were applied. To obtain the number of recruited platelets, the first image of the sequence was extracted, and platelets were counted using the plugin Analyze > Cell counter. To

obtain the number of adherent platelets, pictures of the 30sec sequence were merged in one single image using the command Stacks > Z Project: Sum slices. Thereafter, the image was thresholded using brightness and contrast adjustment to retain only platelets that were immobile on the 30 s sequence. The number of adherent platelets was counted using the Cell counter plugin. All analyses were performed using the Image J software.

Video S1. Streaming movie of a sham-operated mouse acquired using continuous 561 nm singlewavelength stimulation and merged with a 488 nm non-simultaneously acquired movie of the same field of view.

Video S2. Movie of a sham-operated mouse generated by merging sequential acquisitions of the

561 nm and 488 nm channels 15 min after surgery.

Video S3. Movie of a 60% partially hepatectomized mouse generated by merging sequential acquisitions of the 561 nm and 488 nm channels 15 min after surgery.

3. Supplementary references

1. Meyer J, Lacotte S, Morel P, et al. An optimized method for mouse liver sinusoidal endothelial cell isolation. *Exp Cell Res* 2016; 349(2):291-301.
2. Mederacke I, Dapito DH, Affo S, et al. High-yield and high-purity isolation of hepatic stellate cells from normal and fibrotic mouse livers. *Nat Protoc* 2015; 10(2):305-15.
3. Jonnalagadda D, Izu LT, Whiteheart SW. Platelet secretion is kinetically heterogeneous in an agonist-responsive manner. *Blood* 2012; 120(26):5209-16.
4. Bambace NM, Levis JE, Holmes CE. The effect of P2Y-mediated platelet activation on the release of VEGF and endostatin from platelets. *Platelets* 2010; 21(2):85-93.
5. Chatterjee M, Huang Z, Zhang W, et al. Distinct platelet packaging, release, and surface expression of proangiogenic and antiangiogenic factors on different platelet stimuli. *Blood* 2011; 117(14):3907-11.
6. Hoshi R, Murata S, Matsuo R, et al. Freeze-dried platelets promote hepatocyte proliferation in mice. *Cryobiology* 2007; 55(3):255-60.
7. Matsuo R, Ohkohchi N, Murata S, et al. Platelets Strongly Induce Hepatocyte Proliferation with IGF-1 and HGF In Vitro. *J Surg Res* 2008; 145(2):279-86.
8. Battinelli EM, Markens BA, Italiano JE, Jr. Release of angiogenesis regulatory proteins from platelet alpha granules: modulation of physiologic and pathologic angiogenesis. *Blood* 2011; 118(5):1359-69.
9. Aatonen M, Valkonen S, Boing A, et al. Isolation of Platelet-Derived Extracellular Vesicles. *Methods Mol Biol* 2017; 1545:177-188.
10. Kawasaki T, Murata S, Takahashi K, et al. Activation of human liver sinusoidal endothelial cell by human platelets induces hepatocyte proliferation. *J Hepatol* 2010; 53(4):648-54.
11. Hori T, Ohashi N, Chen F, et al. Simple and sure methodology for massive hepatectomy in the mouse. *Ann Gastroenterol* 2011; 24(4):307-318.
12. Jenne CN, Wong CH, Petri B, et al. The use of spinning-disk confocal microscopy for the intravital analysis of platelet dynamics in response to systemic and local inflammation. *PLoS One* 2011; 6(9):e25109.
13. Liu W, Hou Y, Chen H, et al. Sample preparation method for isolation of single-cell types from mouse liver for proteomic studies. *Proteomics* 2011; 11(17):3556-64.

14. Meyer dos Santos S, Klinkhardt U, Schneppenheim R, et al. Using ImageJ for the quantitative analysis of flow-based adhesion assays in real-time under physiologic flow conditions. *Platelets* 2010; 21(1):60-6.
15. Lesurtel M, Graf R, Aleil B, et al. Platelet-derived serotonin mediates liver regeneration. *Science* 2006; 312(5770):104-7.
